# Sex differences in social brain neural responses in autism: temporal profiles of configural face-processing within data-driven time windows

**DOI:** 10.1038/s41598-024-64387-9

**Published:** 2024-06-18

**Authors:** Teresa Del Bianco, Meng-Chuan Lai, Luke Mason, Mark H. Johnson, Tony Charman, Eva Loth, Tobias Banaschewski, Jan Buitelaar, Declan G. M. Murphy, Emily J. H. Jones, The AIMS-2-TRIALS LEAP Team, Simon Baron-Cohen, Sarah Durston, Antonio Persico, Sven Bölte, Antonia San Jose Caceres, Hannah Hayward, Daisy Crawley, Jessica Faulkner, Jessica Sabet, Claire Ellis, Bethany Oakley, Rosemary Holt, Sara Ambrosino, Nico Bast, Sarah Baumeister, Annika Rausch, Carsten Bours, Ineke Cornelissen, Daniel von Rhein, Laurence O’Dwyer, Julian Tillmann, Jumana Ahmad, Emily Simonoff, Joerg Hipp, Pilar Garces, Christine Ecker, Andreas Meyer-Lindenberg, Heike Tost, Carolin Moessnang, Daniel Brandeis, Christian Beckmann, Flavio Dell’ Acqua, Amber Ruigrok, Thomas Bourgeron

**Affiliations:** 1 Centre for Brain and Cognitive Development, Henry Wellcome Building, Birkbeck University of London, Malet Street, London, WC1E 7HX UK; 2 School of Social Sciences and Professions, London Metropolitan University, London, UK; 3 Margaret and Wallace McCain Centre for Child, Youth & Family Mental Health and Azrieli Adult Neurodevelopmental Centre, Campbell Family Mental Health Research Institute, Centre for Addiction and Mental Health, Toronto, ON Canada; 4 Department of Psychiatry, Temerty Faculty of Medicine, University of Toronto, Toronto, ON Canada; 5 Department of Psychiatry, The Hospital for Sick Children, Toronto, ON Canada; 6 Autism Research Centre, Department of Psychiatry, University of Cambridge, Cambridge, UK; 7 Department of Psychiatry, National Taiwan University Hospital and College of Medicine, Taipei, Taiwan; 8 Institute of Psychiatry, Psychology & Neuroscience, King’s College London, London, UK; 9 Department of Psychology, University of Cambridge, Cambridge, UK; 10 Central Institute of Mental Health, Mannheim, Germany; 11 Donders Center of Medical Neurosciences, Radboud University, Nijmegen, The Netherlands; 12 University Medical Center Utrecht, Utrecht, The Netherlands; 13 Università Degli Studi di Messina, Messina, Italy; 14 Department of Women’s and Children’s Health, Karolinska Institutet, Stockholm, Sweden; 15 Hospital General Universitario Gregorio Marañón, Madrid, Spain; 16 Autism Research and Intervention Center of Excellence, University Hospital Frankfurt, Goethe-University, Frankfurt, Germany; 17 University of Greenwich, London, UK; 18 Present Address: F. Hoffmann-La Roche Ltd., Basel, Switzerland; 19 Institut Pasteur, Paris, France

**Keywords:** Social behaviour, Human behaviour

## Abstract

Face-processing timing differences may underlie visual social attention differences between autistic and non-autistic people, and males and females. This study investigates the timing of the effects of neurotype and sex on face-processing, and their dependence on age. We analysed EEG data during upright and inverted photographs of faces from 492 participants from the Longitudinal European Autism Project (141 neurotypical males, 76 neurotypical females, 202 autistic males, 73 autistic females; age 6–30 years). We detected timings of sex/diagnosis effects on event-related potential amplitudes at the posterior–temporal channel P8 with Bootstrapped Cluster-based Permutation Analysis and conducted Growth Curve Analysis (GCA) to investigate the timecourse and dependence on age of neural signals. The periods of influence of neurotype and sex overlapped but differed in onset (respectively, 260 and 310 ms post-stimulus), with sex effects lasting longer. GCA revealed a smaller and later amplitude peak in autistic female children compared to non-autistic female children; this difference decreased in adolescence and was not significant in adulthood. No age-dependent neurotype difference was significant in males. These findings indicate that sex and neurotype influence longer latency face processing and implicates cognitive rather than perceptual processing. Sex may have more overarching effects than neurotype on configural face processing.

## Introduction

Autism is a neurodevelopmental condition characterized by differences in social and sensory-motor behaviours, with consequences for daily activities and mental health^1^, with an estimated prevalence 1–2%^2^. This rate varies by sex assigned at birth, with the prevalence in males 3.8 times higher than females^3^.

To identify the neurocognitive mechanisms associated with sex differences, a range of studies have focused on visual attention. Studies where the majority of autistic participants are male have shown an average reduction of the visual preference for faces—termed social attention^4^—compared with neurotypical peers^5^. Eye-tracking studies showed that divergent gaze patterns emerge early^6^, with less attention to faces compared with neurotypicals^7^, but with heterogeneity that has led to mixed and null replications^8^. Nevertheless, variations in these differences are influenced by sex and/or gender, with female autistic and neurotypical individuals showing smaller average differences in visual social attention compared to the differences observed between autistic and neurotypical males^9–11^. These on-average differences might result from joint effects of sex-related and gender-related factors on the manifestations and recognition of autism^12,13^.

Findings from large samples showed that differences in the timings of social attention between autistic and neurotypical people have larger effect sizes than averaged looking times^14^, and also interact with age and sex^10^. For example, autistic females show distinct temporal profiles of social attention in adulthood, despite neurotypical-like average-looking times to faces^10^.

However, eye-tracking studies, while valuable, only provide insights to behavioural responses to particular stimuli and tasks that may vary with sex and/or gender, without delving into the underlying neural mechanisms. Electroencephalogram (EEG) complements this by offering additional information on neural processes and their temporal sequence involved in social attention. Specifically, the capability of EEG to detect event-related potentials (ERPs), which occur rapidly on the scale of milliseconds, facilitates the identification of neural responses specific to stimuli and tasks where individual variations and interactions may be related to factors like sex and/or gender, thereby providing a more fine-grained understanding of social attention. Most importantly, EEG enables the distinction between early and late neural responses towards stimuli such as faces, that may represent different underlying neural processes^14^. This differentiation is crucial for understanding the dynamics of attentional processes and how they manifest at the behavioural level in social attention.

Configural face processing (i.e., integration of facial features that facilitates recognition^15^) in autism has been studied with ERPs for decades. On average, autistic people show divergent early- and late-stage neural activities in response to faces compared with alternative stimuli^16^. This phenomenon can be recorded in the early-occurring ERPs, such as the N170, a face-specific neural response occurring 170 ms post-stimulus, primarily recorded at lateral posterior electrodes P7 and P8, that reflects the activity of the neural networks underlying face-processing^17^. The N170 is more negative in response to inverted faces, reflecting perceptual discrepancy^18^ and face specialization^19^. Autistic people overall show less pronounced face-inversion effects compared with neurotypical peers on the N170, with smaller differences in amplitude between upright and inverted faces^20,21^. This could stem from socio-communication features of autism that render autistic people less exposed to faces in daily life^21^ and/or early-stage processing differences between autistic and non-autistic people that lead to greater difficulty with processing faces^22^. Each of these mechanisms (independently, co-existing, or feeding into each other) could have cascading effects on the smoothness and success of interpersonal interactions that rely on face-processing abilities^22^.

In the general population, face processing varies by sex, such as faster and higher amplitudes before 250 ms post-stimulus onset to faces compared to objects^23,24^ and enhanced face-inversion effects^25^ in females compared to males. These findings may reflect enhanced early-stage processing of faces in females, a pattern compatible with eye-tracking findings of greater attention deployment to faces in females^26^. These on-average differences might have a biological explanation as supported by evidence showing that testosterone level^27^ and the menstrual cycle^28^ influence social attention and face recognition in macaque rhesus monkeys. However, there may also be influences of socialisation pressure including gender socialisation, particularly in females, as evidenced by larger effects sizes of sex-differentiated visual preferences in females in self-reports compared to eye-tracking that may be due to internalised expectations^29^, and the observation that female children (but not male children) associate gender-stereotyped toys with girls’ and boys’ faces, despite both sexes display stereotyped preferences in play^30^.

Investigations with EEG of sex influences on face processing in autistic people are relatively rare^31^; similarly to the general population and as outlined above, exploratory findings suggest that neural face-processing patterns in autistic females are associated with phenotypes such as social responsiveness, communication and social interaction^19^. Therefore, a dedicated investigation of sex-differentiated patterns of face processing in autism may prove useful. This investigation verify whether the same tendencies as the general population may be found, and whether they relate to neural responses that are more likely to be influenced by biological and/or socialisation factors respectively (e.g., early deflection of potential indexing automatic detection of faces versus late inflection of potential indexing higher order processing and memorisation).

A recent analysis of a sample with clinically-observed autism male-to-female ratio (3:1^32^)—the Longitudinal European Autism Study^33,34^—found no statistically significant interaction between diagnosis/neurotype and sex in N170^20^. However, standard ERP methods rely on the detection of single features at one timepoint, and this null result might reflect insufficient sensitivity in such a single feature to detect sex-modification effects, a limitation that also applies to eye-tracking average vs temporal profiles in the same cohort^10,14^. On this premise, we set out the current analyses, where we applied a data-driven approach to ERPs in response to upright and inverted faces, to detect time-windows that are sensitive to sex differences in autistic and non-autistic people. Specifically, we developed a two-stage analysis: bootstrapped cluster-based permutation analysis (BCPA) of the ERPs, and growth curve analysis (GCA) of the face-inversion effect.

Different from standard ERP methods, BCPA is a single-channel method that maximises temporal resolution and sensitivity to specific processing; as such, it does not pre-determine a specific timepoint for the detection of a single feature and can obtain information of the *timing* (i.e., onset and offset) based on the internal correlations of time-bins^35–38^. As single channel, we selected P8, due to its maximal sensitivity to face processing^20,39–42^. Based on previous findings of case–control analysis between males and females, and between autistic and non-autistic people^19,31^, we expected the onset and offset of the effect of sex to be within the first 250 ms after stimulus onset (but not necessarily overlapping with the N170). We also hypothesized that the timing of this effect would precede the timing of the effect of neurotype^9,43^. This prediction is justified by the evidence that sex influences the precursors of the manifestations of autism^22^, putatively by means of an early bias towards faces in females that modifies neurodivergent phenotypes^22^. Additionally, we repeated this analysis on subsets of female, male, autistic and non-autistic individuals only; this approach allows for estimating each effect taking the subset-average as a reference, rather than the whole sample average. This approach could help (a) to reliably estimate the timing of the effect of neurotype in the smaller female subsample; and (b) to interpret large differences between male and female, and between autistic and non-autistic individuals that might be deflated when the whole sample is pooled together. The time-clusters obtained will indicate when systematic differences are larger for a given subgroup, e.g., the differences between autistic and non-autistic females. In line with our preceding hypothesis, we expected such differences to be stronger earlier than later in the trial considering the enhanced automatic processing of faces in females.

Second, we employed GCA to delve into the neurophysiological temporal profile of the face-inversion effect within the broader time-cluster detected through data-driven analysis. By utilising GCA, we aim to explore how this temporal profile varies across different demographic variables such as sex and neurotype, as well as age-groups. Unlike traditional multivariate methods such as ANOVA, GCA offers the advantage of examining differences in the time-series data rather than focusing on overall differences^14,44^. This allows for a nuanced understanding of how the temporal dynamics of neural responses to face inversion may differ across different populations. In line with previous findings, we expected:

- Neurotypical females to exhibit enhanced face-inversion effects, with the direction of effect depending on the onset and offset of the time-cluster (e.g., positive difference in N170, but negative for later components such as the P300 that indexes novelty detection^45^).
- Enhanced face-inversion effects in neurotypical females compared to autistic females, and neurotypical and autistic males (in descending order)^19^.
- Stronger differences in the cubic component of the GCA, with a significantly positive cubic (i.e., a peak followed by a trough) in neurotypical females compared with the other sex/neurotype combinations. The cubic component describes two significant inflection of potential corresponding to 3 changes of stage (e.g., sensory reception, integration and processing, and perceptual and cognitive outcomes such as attention and memorisation^44^).
- These differences to differ between children, adolescents and adults, due to the progressive maturation of face specialization; based on previous findings, adults should be more specialised in face processing and thus display the largest face-inversion effect compared to younger participants^46^.

## Results

### Bootstrapped cluster-based permutation analysis

The characteristics of the participants are reported in Table 1.

**Table 1:** Descriptive statistics of the analysis sample.

	Neurotypical (NT)				Autistic (A)			
Sex assigned at birth	Male (M)	Female (F)			Male (M)	Female (F)		
Number	141	76			202	73		
N FSIQ < 75	18	11			36	17		
Age Range (Min–Max), years	7.53–30.98	6.89–30.78			6.57–29.88	6.08–30.28		

			Difference effect size				Difference effect size	
			NT M vs NT F	NT M vs A M			A M vs A F	NT F vs A F
Amplitude at P8 to inverted faces	− 0.15 (0.85)	0.08 (1.02)	0.24	0.03	− 0.12 (1.04)	− 0.07 (0.94)	0.07	0.15
Amplitude at P8 to upright faces	0.06 (0.94)	0.37 (1.05)	0.31	0.14	− 0.08 (1.01)	− 0.07 (0.94)	0.01	0.44
Amplitude difference between upright and inverted faces	0.20 (0.81)	0.29 (0.86)	0.10	0.16	0.06 (0.86)	0.34 (0.87)	0.32	0.05
Age mean (SD), year	17.84 (5.69)	16.48 (5.72)	0.23	0.14	17.02 (5.3)	16.88 (6.28)	0.02	0.09
FSIQ mean (SD)	101.4 (19.13)	103.24 (19.31)	0.09	0.28	95.85 (20.09)	93.5 (19.24)	0.11	0.50
ADOS CSS total mean (SD)	–	–	–	–	5.89 (2.73)	4.33 (2.56)	0.58	–
SRS-2 total T-score mean (SD)	48.45 (9.1)	47.28 (7.8)	0.14	2.07	70.29 (11.74)	71.45 (12.95)	0.09	2.26
Eye-tracking individual random effects to Face pop-out task-intercept*	0.20 (0.06)	0.24 (0.08)	0.56	0.001	0.20 (0.07)	0.22 (0.09)	0.24	0.23
Eye-tracking individual random effects to face pop-out task-slope*	− 0.15 (0.14)	− 0.14 (0.18)	0.06	0.001	− 0.14 (0.16)	− 0.15 (0.16)	0.06	0.05
Eye-tracking individual random effects to face pop-out task-quadratic*	0.13 (0.11)	0.17 (0.12)	0.34	0.33	0.09 (0.13)	0.13 (0.14)	0.29	0.30
Eye-tracking individual random effects to face dynamic video task-intercept*	0.72 (0.12)	0.77 (0.10)	0.45	0.16	0.66 (0.14)	0.70 (0.12)	0.30	0.63
Eye-tracking individual random effects to face dynamic video task-slope*	0.24 (0.08)	0.26 (0.07)	0.26	0.44	0.20 (0.10)	0.21 (0.08)	0.11	0.66
Eye-tracking individual random effects to face dynamic video task-quadratic*	− 0.28 (0.07)	− 0.32 (0.04)	0.70	0.53	− 0.24 (0.08)	− 0.29 (0.06)	0.70	0.58

FSIQ < 75 is considered a threshold for intellectual disability^61^. Difference effect sizes between means calculated as Cohen’s D^62^.

*For full details on the eye-tracking individual random effects and face pop-out and dynamic videos eye-tracking tasks, see^10^.

**Figure 1:**
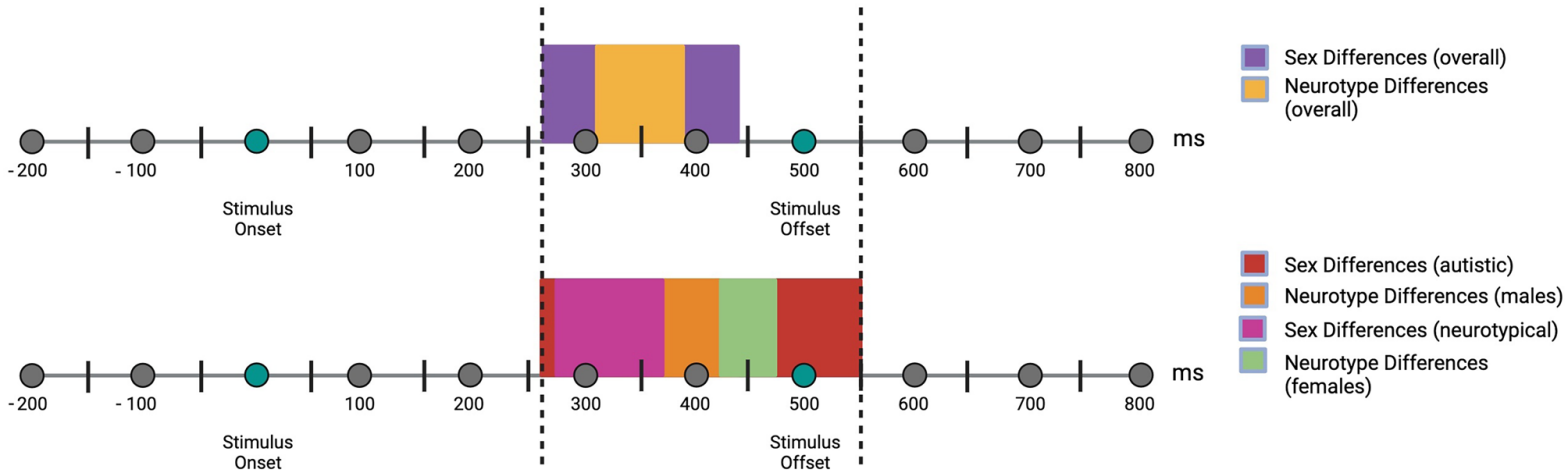
Onset and offsets of the time-clusters listed in Table 2.

To estimate when the interaction between condition and neurotype occurred in the time course of face processing, we tested the grouping of adjacent time-bins with BCPA conditioning on sex or using sex as a covariate at the channel that showed the stronger faces-sensitive response, P8 (see electrode and hemisphere analysis and Fig. 1C in Ref.^20^). The onset of the interaction between sex and condition was earlier than the interaction with neurotype—260 ms (Beta =  − 0.29, SE = 0.01, p-value = 0.03) vs 310 ms (Beta = 0.17, SE = 0.08, p-value = 0.02), while the offset was later—440 (Beta =  − 0.004, SE = 0.001, p-value < 0.001) vs 390 (Beta = 0.16, SE = 0.07, p-value = 0.01). In all subgroups, the onset of the interaction with sex occurred earlier than neurotype, as well as offsetting later; of note, the latest onsetting interaction was in females between condition neurotype, significant between 430 (Beta =  − 1.05, SE = 1.22, p-value < 0.001) and 480 ms (Beta = 0.90, SE = 1.17, p-value < 0.001). See Table 2 and Fig. 1 for all onsets and offsets, and Table S1 of the SM for all effect sizes/Beta, SE and p-values.

**Table 2:** Onset and offset of the data-drive time-clusters.

Effect	Neurotype			Sex		
Subgroup	All	Female	Male	All	Autistic	Neurotypical
Onset–offset	310–390	430–480	310–450	260–440	260–550	280–370

### Growth curve analysis

**Figure 2:**
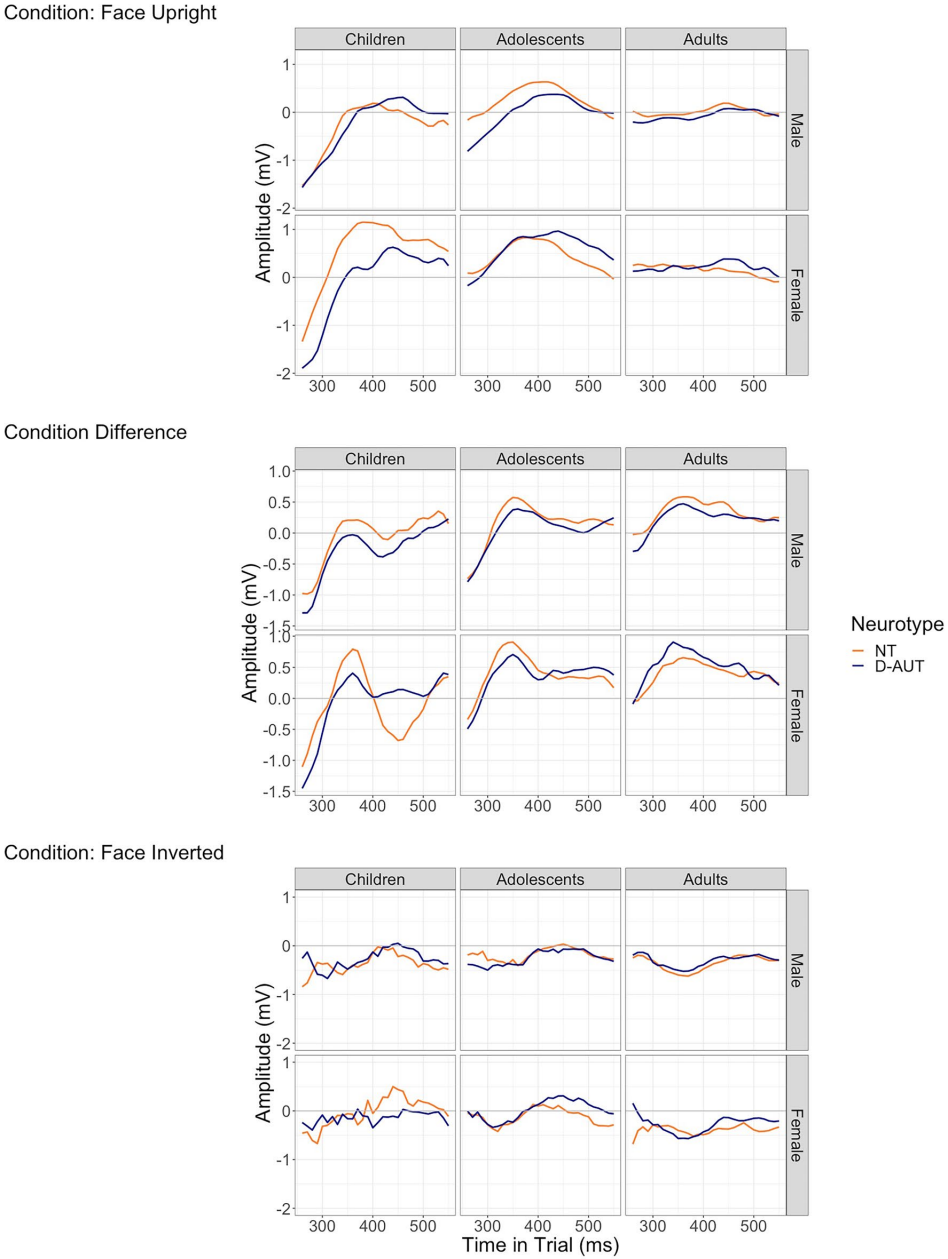
Change of amplitude in the two conditions (top and bottom plots), and difference between the two conditions (middle plot) at P8. In the middle plot, the amplitude forms a peak followed by a trough (i.e., an approximated Z-shape plotted horizontally, accentuated in the neurotypical female children) corresponding to the significant positive cubic component found in the GCA.

With GCA, we examined the neurophysiological temporal profile of the face-inversion effect (i.e., the difference between amplitudes in the upright minus the inverted condition) within the wider detected data-driven time-cluster—260 ms (earliest onset of a time cluster) to 550 ms (latest offset of a time cluster) post-stimulus onset—and how it varies between sexes, neurotypes, conditions (upright/inverted) via interactions, and age via conditioning (to avoid a 4th-way interaction). By age and neurotype sex differences are fully reported in Table 3, and plainly summarised in Table 4. Below, we describe the timecourse of statistically significant differences in detail. The temporal profiles found in the data and analysed here are visually represented in Fig. 2, mid-panel.

**Table 3:** Coefficient estimates, standard errors (SE), t-values, degrees of freedom (df), p-values of the GCA mixed regression models.

Children					
Term	Coefficient estimate	SE	T-value	df	P-value
Intercept	− 9.79	1.49	− 6.59	546.07	< 0.001
Linear component (slope)	165.63	23.75	6.97	2390.11	< 0.001
Quadratic component	− 84.47	13.42	− 6.29	558.13	< 0.001
Cubic component	55.68	8.93	6.23	2479.99	< 0.001
Neurotype (autistic)	− 1.20	1.85	− 0.65	546.07	0.52
Sex (female)	− 12.60	2.19	− 5.76	546.07	< 0.001
Linear component (slope) × neurotype (autistic)	26.59	29.59	0.90	2390.13	0.37
Quadratic component × neurotype (autistic)	− 8.71	16.72	− 0.52	558.13	0.60
Cubic component × neurotype (autistic)	11.60	11.13	1.04	2479.99	0.30
Linear component (slope) × sex (female)	252.48	34.96	7.22	2390.14	< 0.001
Quadratic component × sex (female)	− 116.61	19.76	− 5.90	558.13	< 0.001
Cubic component × sex (female)	104.08	13.15	7.92	248 < 0.001	< 0.001
Neurotype (autistic) × sex (female)	8.39	3.02	2.78	546.07	0.01
Linear component (slope) × neurotype (autistic) × sex (female)	− 186.31	48.20	− 3.87	2390.15	< 0.001
Quadratic component × neurotype (autistic) × sex (female)	77.07	27.24	2.83	558.13	< 0.001
Cubic component × neurotype (autistic) × sex (female)	− 82.50	18.13	− 4.55	248 < 0.001	< 0.001
Adolescents					
(Intercept)	− 11.04	0.69	− 15.93	1122.84	< 0.001
Linear component (slope)	182.03	11.09	16.42	4815.07	< 0.001
Quadratic component	− 99.05	6.12	− 16.18	1309.13	< 0.001
Cubic component	62.02	4.16	14.90	5017.94	< 0.001
Neurotype (autistic)	0.34	0.90	0.37	1122.82	0.71
Sex (female)	− 0.01	1.13	− 0.01	1122.80	0.99
Linear component (slope) × neurotype (autistic)	− 1.26	14.39	− 0.09	4815.00	0.93
Quadratic component × neurotype (autistic)	3.91	7.94	0.49	1309.10	0.62
Cubic component × neurotype (autistic)	0.74	5.40	0.14	5017.96	0.89
Linear component (slope) × sex (female)	10.98	18.14	0.61	4814.95	0.55
Quadratic component × sex (female)	− 4.05	10.02	− 0.40	1309.08	0.69
Cubic component × sex (female)	7.61	6.81	1.12	5017.97	0.26
Neurotype (autistic) × sex (female)	2.38	1.59	1.49	1122.79	0.14
Linear component (slope) × neurotype (autistic) × sex (female)	− 45.42	25.49	− 1.78	4814.92	0.07
Quadratic component × neurotype (autistic) × sex (female)	22.45	14.07	1.60	1309.06	0.11
Cubic component × neurotype (autistic) × sex (female)	− 19.80	9.57	− 2.07	5017.98	0.04
Adults					
(Intercept)	− 5.45	0.51	− 10.78	1102.18	< 0.001
Linear component (slope)	87.12	8.03	10.84	4887.84	< 0.001
Quadratic component	− 51.50	4.52	− 11.40	1194.59	< 0.001
Cubic component	28.66	3.01	9.51	5126.04	< 0.001
Neurotype (autistic)	− 0.81	0.68	− 1.19	1102.19	0.23
S ex (female)	− 0.14	0.93	− 0.15	1102.20	0.88
Linear component (slope) × neurotype (autistic)	16.70	10.78	1.55	4887.86	0.12
Quadratic component × neurotype (autistic)	− 5.84	6.06	− 0.96	1194.60	0.34
Cubic component × neurotype (autistic)	6.41	4.04	1.58	5126.02	0.11
Linear component (slope) × sex (female)	5.14	14.81	0.35	4887.90	0.73
Quadratic component × sex (female)	− 1.76	8.33	− 0.21	1194.61	0.83
Cubic component × sex (female)	1.94	5.56	0.35	5126.01	0.73
Neurotype (autistic) × sex (female)	− 1.16	1.26	− 0.92	1102.20	0.36
Linear component (slope) × neurotype (autistic) × sex (female)	16.87	20.01	0.84	4887.90	0.40
Quadratic component × neurotype (autistic) × sex (female)	− 13.46	11.24	− 1.20	1194.61	0.23
Cubic component × neurotype (autistic) × sex (female)	6.40	7.50	0.85	5126.00	0.39

**Table 4:** Significant effects of the GCA and direction of the effects.

Age group	Average inversion effect	Cubic component
Children	Neurotypical females: more negative inversion effects compared to neurotypical males Autistic females: more positive inversion effect than neurotypical females	Neurotypical females: more positive cubic component compared to neurotypical males Autistic females: more negative cubic component than neurotypical females
Adolescents	Autistic females: more positive inversion effect than neurotypical females	Autistic females: more negative cubic component than neurotypical females
Adults	No differences	No differences

In children, the difference between conditions was significantly negative on average (Intercept Coefficient Estimate =  − 9.79, SE = 1.49, p-value < 0.001), and formed a significant positive wave followed by a negative wave (Cubic Coefficient Estimate = 55.68, SE = 8.93, p-value < 0.001). Female sex had a significant effect on the average difference between conditions, that was more negative (Intercept × Sex Coefficient Estimate =  − 12.60, SE = 2.19, p-value < 0.001), with an accentuated significant peak followed by a trough (Cubic Coefficient Estimate = 104.08, SE = 13.15, p-value < 0.001). The significant negative average difference indicates that over the whole time-cluster, the amplitude was more negative for upright faces. However, a significant peak followed by a trough indicates that this difference between conditions changes in time: starting with more negative amplitudes for upright faces compared to inverted, followed by more positive amplitude for upright faces compared to inverted, again followed by more negative amplitudes for upright faces compared to inverted (see Fig. 2).

The interaction between neurotype and sex was significant, meaning that autistic females showed an overall less negative difference (Neurotype × Sex Coefficient Estimate = 8.39, SE = 3.02, p-value = 0.01) and less positive cubic (Cubic Coefficient Estimate =  − 82.50, SE = 18.13, p-value < 0.001), indicating an attenuated peak followed by a trough.

In adolescents, the difference between conditions was significantly negative on average (Intercept Coefficient Estimate =  − 11.04, SE = 0.69, p-value < 0.001), with a significant peak followed by a trough (Cubic Coefficient Estimate = 62.02, SE = 4.16, p-value < 0.001), with no difference by sex or neurotype. The only significant interaction between neurotype, sex and the cubic component of the GCA indicated an attenuated peak followed by a trough in autistic females (Cubic Coefficient Estimate =  − 19.80, SE = 9.57, p-value = 0.04).

In adults, the difference between conditions was significantly negative on average (Intercept Coefficient Estimate =  − 5.45, SE = 0.51, p-value < 0.001) and formed a significant peak followed by a trough (Cubic Coefficient Estimate = 28.66, SE = 3.01, p-value < 0.001), with no significant difference by sex or neurotype, and no significant interactions. The full list of estimates, standard errors, t-values, degrees of freedom, and p-values are available in Table 3. The direction of the significant results (positive or negative) is summarised in Table 4.

### Exploratory pairwise correlations

The only significant correlations after correction for multiple comparisons were between the FSIQ and the average estimated face-inversion effect (rho = 0.32, corrected p-value = 0.02) and its slope (rho =  − 0.36, corrected p-value = 0.01) in neurotypical females. No other correlation was significant, and all were weak (< 0.30) in size (for the full list of rhos and p-values, see Supplementary Material, Tables S11–S15).

**Figure 3:**
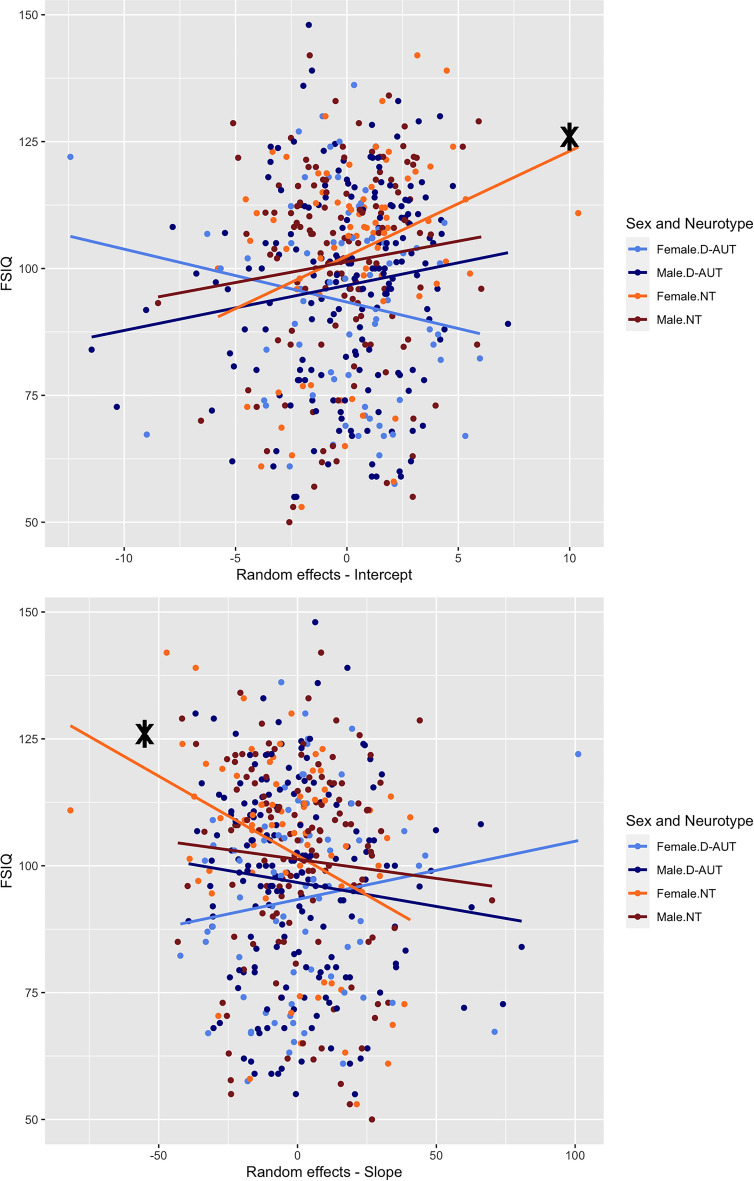
Scatterplot of FSIQ and the individual random effects of Intercepts and Slopes. The significant correlation between these variables in the neurotypical females is marked with a capitalised “*”.

The two significant correlations indicate that among neurotypical females, those with higher FSIQ tend to exhibit an average face-inversion effect (intercept) that is higher than the overall effect estimate. Additionally, their trajectory (slope) shows a downward trend compared to the overall effect estimate. In other words, in neurotypical females with higher FSIQ, the magnitude of the face inversion effect is larger and remains consistently significant throughout the trial duration (see Fig. 3).

## Discussion

The aim of this study was to investigate whether differences in the timecourse of neural responses to faces vary by sex and neurotype, examining both overlaps and discrepancies. To detect time-windows of neural responses that are sensitive to sex differences among autistic and non-autistic people, we segmented event-related potentials in response to upright and inverted images of a human face with a data-driven approach.

Our initial hypotheses based on previous findings are only partially supported. We found that the timing of the sex effect precedes the timing of the effect of neurotype, but they both occurred after the first 250 ms post-stimulus onset. Therefore, the time-window where sex differences are stronger in this sample does not involve those that have been previously reported in connection with an early bias towards faces in females in the general population, such as the N170^23^.

When looking at specific groups of interest, we again found significant clusters onsetting later than expected, after visual and perceptual analysis of faces^15^. First, in the female subsample, neurotype differences were stronger closer to the end of trial, after 430 ms post-stimulus onset, a time-window associated with familiarisation to individual faces^47^. Sex differences in neurotypical individuals and neurotype differences in male individuals were stronger in the earlier portion of the data-driven time-cluster (280–370 ms for neurotypicals, and 310–450 ms for males), when processes related to novelty and information processing might occur^48,49^. Differences in this time-window might have overshadowed later-onsetting neurotype differences in females in previous studies with smaller female samples.

With age-conditioned growth curve analysis, we examined the temporal profile of the face-inversion effect within the detected time-clusters. Between 260 and 550 ms post-stimulus (the earliest onset and latest offset of the data-driven time-clusters), the estimated face-inversion effect was negative across all age groups, indicating more negative amplitudes for upright faces on average, and more positive amplitudes for inverted faces. As predicted, the temporal profile of the face-inversion effect fitted the positive cubic component of the GCA, indicating that the face-inversion effect changed across time following an approximate sinusoidal shape. These results partially fit our predictions, with a few unexpected findings. First, in line with our predictions, neurotypical females showed an enhanced (more negative) face-inversion effect, and a more positive cubic component of the GCA compared to all other subgroups, but this was only significant in children aged 6 to 11 years. Adolescent and adult neurotypical females did not show such an enhanced face-inversion effect compared to all other subgroups. Female autistic children showed an intermediate face-inversion effect (more negative than autistic and neurotypical males, but less than neurotypical females), and a significantly attenuated cubic component/sinusoidal shape compared with neurotypical females. This difference diminished in autistic female adolescents (with only significantly attenuated cubic) and became non-significant in female adults. This within-female difference in the cubic component of the GCA involving children to adolescents included the late trial after 400 ms, when neurotypical females’ sinusoidal shape face-inversion effect became negative again, while mostly flat in autistic females. This was also the segment where neurotype differences were stronger between females according to the data-driven time-clustering. Exploratory correlations revealed that the enhanced face-inversion effect in neurotypical females was positively associated with FSIQ.

These results are compatible with evidence of maturation of neural specialisation towards faces during childhood, represented by ERPs even after 250 ms. The enhancement observed in female children supports the idea that early in life females invest more neural resources towards face specialisation, a process that could be explained with a mix of predispositional, hormonal, environmental, and socialization pressure. Echoing our eye-tracking findings, where the same autistic girls showed a later peak looking to faces compared with neurotypical girls^10^, autistic girls showed an attenuated enhancement of the face inversion. Intriguingly, all differences receded with age, with autistic females showing patterns more alike neurotypical females by adulthood, and males and females of either neurotypes showing the same average values and temporal profiles. It seems that, unlike other sex differentiated components such as visual preference^50^, maturation here results in diminished differences and more similar face-inversion effect. The enhancement in female children might signify that they use more neural resources in face processing compared to male children. The positive correlation between our index of face processing and FSIQ aligns with the evidence in neurotypical populations^51^ and supports this explanation, as it might be the enhanced facial recognition abilities and cognitive processes that render neurotypical females more adept at processing complex visual information (such as inverted faces).

In either case, these findings highlight the importance of considering sex as a modifier in understanding the neurocognitive processing of autistic individuals and underscore its unique effects on the neural processing in children. We were able to detect such effects by focusing on a data-driven time-cluster that does not contain responses of automatic orienting and speed of processing, that have been considered a female advantage^52^. Rather, the time-cluster between 260 and 550 ms contains processes related to sustained attention and face information processing^53,54^ that may support female-related cognitive styles (e.g., wider social networks^55^) and socialization pressure (e.g., reward value of intimacy and interactions^56^). For autistic female individuals (and even more if undiagnosed and unsupported), with specific sensory and alertness needs, these processes may represent a double-edged sword: enabling the accumulation of expertise, but also exposing to the risk of generalised drain, episodic meltdowns and burn out^57,58^. Future studies should investigate the correlates of the diminishment of the effect between autistic and neurotypical teenagers and adults in neurophysiological indices and verify whether it correlates with masking and mental health consequences.

This study includes a series of limitations including cross-sectional design that prevents direct modelling of developmental trajectories, static stimuli that do not provide dynamic facial expressions and context, a smaller female sample size compared to the males, and lack of information on gender identity and gender socialization effects. Thus, replication with independent samples and with larger female representation will improve the generalizability of the findings. Furthermore, we focused on P8 based on previous literature^20,41^ and supported by the topography recorded in this sample (see Fig. S1 in the SM). Prior research within our sample indicates that there were no group differences in topography of EEG responses to faces across the studied timewindow^59^. Moreover, we did not employ source localization techniques and thus the spatial spread of neural activity captured by P8 may encompass very close locations such as PO7/8, accommodating subtle differences^60^. However, in the light of our findings, the area of interest could include other parietal and central electrodes^61^, such as P08 and TP10, PO2, POz, PO4. Of note, averaging across electrodes might introduce additional variance and lead to the loss of timeframes of interest, as demonstrated in our replication analysis detailed in the SM (section ‘Replication of Bootstrapped Cluster-based Permutation Analysis on Averaged Electrode Clusters’). Additionally, electrodes across hemispheres^62,63^, like P7 and PO7, may provide an earlier onset of timeframes closer to the N170 (see SM). However, averaging across electrodes may come with increased variance that could obscure the detected effect and extend the duration of the timeframe well beyond components of interest, such as the N170. We did not find concurrent associations between EEG parameters and behaviour phenotypes, which might mean that the available clinical measures do not include constructs modified by differential neural responses to faces, or that they might occur before 6 years of age. The choice of clinical metrics and the ages at which they are collected would benefit from consultation and co-design with autistic people, as their insights can inform the selection of the most sensitive measures for the strategies they employ for face processing and social attention. For instance, we found that traditional clinical metrics such as the Social Responsiveness Scale (SRS) and the Autism Diagnostic Observation Schedule (ADOS) did not correlate with our measure of face processing. This observation suggests that these metrics may not capture the behaviours that autistic individuals use to specialise in face processing, at least within the age range (6 to 30 years) of our sample. By actively involving autistic individuals in the research process, we can gain insights into the behaviours and strategies they employ, as well as at what ages these are most relevant. This information can in turn help inform the selection of more meaningful measures for assessing face specialisation and social attention, and determine the appropriate ages to measure them. EEG parameters did not correlate with eye-tracking in another session, despite the observed EEG differences occurring after 250 ms, which aligns with the average latency of mature saccades. However, concurrent eye-tracking and EEG measurements are needed to properly investigate their connections^64^. From the ERP perspective, while differential fixations after 200 ms may contribute to the results, they may not explain them entirely since trials contaminated by eye-movements were excluded during pre-processing^20^.

In conclusion, we show that sex and neurotype EEG differences might be clearest during the later stages of face processing. We found that differences in amplitude after 200 ms were stronger in female children (more in neurotypical, less in autistic) and attenuated with age. This pattern is consistent with eye-tracking findings in the same cohort where female children differed by neurotype in visual attention, whose levels became more similar at older ages^10^. The real-life implications of these findings need to be better understood with concurrent measures of EEG, eye-tracking, and associated behavioural and mental health factors.

## Methods

### Participants

The initial sample consisted of 544 participants of LEAP across 5 sites that collected EEG: King’s College London (KCL, United Kingdom), University Medical Centre Utrecht (UMCU, Netherlands), Radboud University Medical Centre (RUMC, The Netherlands), Mannheim Central Institute of Mental Health (CIMH, Germany), and the University Campus Bio-Medico in Rome (UCBM, Italy)^33^. Independent site ethics committee (KCL: London Queen Square Health Research Authority Research Ethics Committee; RUNMC and UMCU: Radboud Universitair Medisch Centrum Instituut Waarborging Kwaliteit en Veiligheid Commissie Mensgebonden Onderzoek and Regio Arnhem-Nijmegen; CIMH: UMM Universitatsmedizin Mannheim, Medizinishe Ethik Commission II; UCBM: Comitato Etico Università Campus Bio-Medico Di Roma) approved the study at each independent site, and the produces followed the principles of Good Clinical Practice (ICH GCP)^34^. Written informed consent was obtained from the participants and parent/legal guardian where appropriate. The final sample after pre-processing included 492 participants (Table 3).

### Materials

64-channels EEG caps with 10–20 layout at 5000 Hz samplite rate were used. From all processed data, we used amplitudes from P8 at which face processing activity is at its strongest^20,35,36^) following the requirements of single-sensor BCPA^35^.

#### Clinical measures

For the exploratory pairwise correlations, we used three clinical measures:

- Full-Scale Intelligence Quotient (FSIQ): measured with the Wechsler Abbreviated Scale of Intelligence 2nd Edition (WASI-II) in the UK^65^, with the short form of the WISC-III/IV in Germany and the Netherlands^66^, and WAIS-III/IV in Italy^65^. National norms were used to derive standard estimates of FSIQ. To harmonise across sites, the data were pro-rated across 2 verbal and 2 performance subtests to estimate one highly correlated final score^33^.
- Social Responsiveness Scale (SRS-2) Total T-Score: parent/self (adults) reported questionnaire of autism-related socio-communication and restricted-repetitive behaviours, normed by sex and age^67^.
- Autism Diagnostic Observation Schedule (ADOS) Calibrated Severity Score (CSS) of the total score: clinician-reported measure of symptoms associated with autism (i.e., the total score across Social Affect and Restricted and Repetitive Behaviour domains), calibrated by modules. This was only available for autistic participants^68^.

#### Eye-tracking individual random effects

For the exploratory pairwise correlations (see “Statistical analysis” section), we used eye-tracking individual random effects derived from the models described in Ref.^10^ that showed significant sex differences (during the Face Pop Out and the Dynamic Video). The hypothesis here is that in females, face-inversion effects would significantly correlate with metrics that show significant sex differences (e.g., positive correlations between face-inversion effects and eye-tracking individual random effects). The random effect represents the individual differences in social attention at the average level (intercept) and in the temporal profile (slope and quadratic components of the GCA).

### Stimuli

Three 13-cm width upright and inverted faces (passport-like photographs of one Asian, one Black, and one White woman with neutral expressions, taken from the NimStim set^69^), were presented as two conditions at the centre of a grey background harmonised in size between sites. Colourful schematic objects (4-cm width) were presented between trials over the eye region of the face.

### Procedure

**Figure 4:**
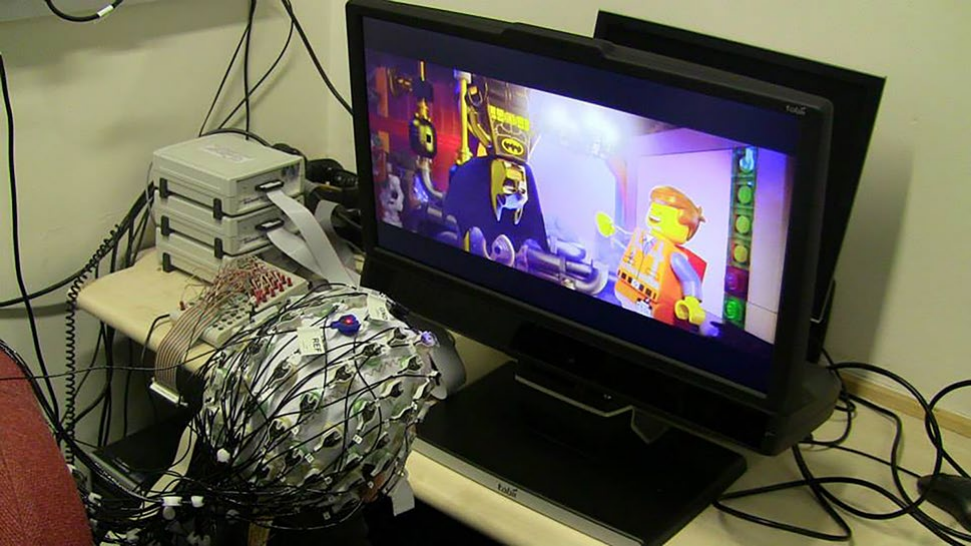
An example of set-up and stimuli used in the experiment.

Participants sat ~ 60 cm away from the screen and watched a distracting video while the experimenter placed the EEG cap on their head and filled electrolyte gel inside the electrodes (Fig. 4). Stimuli were presented with the collection of MATLAB functions *TaskEngine*^20^ or Presentation® software (Neurobehavioral Systems, Inc., Berkeley, CA, www.neurobs.com) at University Medical Centre Utrecht. Faces were presented for 500 ms 168 times over 40 trials in 4 blocks (4.5 min) interspersed with 3 tasks (27.5 min). Between faces, a 350-ms blank grey screen and an inter-trial fixation object (at a random interval between 500 and 700 ms) were presented.

### Pre-processing

The detailed pre-processing steps are described in Ref.^20^. In brief, pre-processing included correction of time delays between stimulus presentation and markers with a screen photodiode, segmentation between − 200 and 800 ms after stimulus onset (i.e., 1 trial), bandpass filter (0.1–40 Hz), FFT-based DFT notch filter (50 Hz), resampling to 500 Hz with padding of 2 s, automatic blinks, eye-movements and whole scalp removal and interpolation. For our chosen single-sensor, P8, we further excluded time-bins with less than 20 trials (91.97% retention in the autistic group, 91.17% retention in the neurotypical group), which resulted in a final dataset of 963,840 rows with a resolution of 0.001 s, that were aggregated into 10 ms time-bins. Finally, we filtered out the first and the last time-bins, which accounted for only 1 and 3 ms, respectively.

### Statistical analysis

We performed Bootstrapped Cluster-based Permutation Analysis in R version 4.1.0 with function *clusterperm.lmer* from package *permutes* version 2.2. This method draws random data samples and calculates a single test statistic, thus avoiding multiple comparisons^35^. With *clusterperm.lmer,* the test statistic and p-value were calculated with mixed-effects regression models.

#### Bootstrapped cluster-based permutation analysis (BPCA)

We ran BCPA on the whole sample and on subgroups with the r function clusterperm.lmer from package permute^70^. The function performs 1000 permutations. The dependent variable was amplitude at P8 for the whole duration of the trial (i.e., up to 800 ms), to maximise the statistical power (i.e., testing critical windows against all possible windows) and look at different stages of face processing. The fixed effects were sex (female/male), neurotype (autistic/neurotypical) in interaction with condition, with correlated random effects per participant by condition (see Eq. 1). The subgroups were female/male participants, and autistic/neurotypical participants. From the whole sample, and the subgroups, we obtained 6 estimates of the onsets and offsets of the interaction effect. For each, we report the effect size (termed beta), standard error (SE) and p-value.

1 $${y}_{i/C}= {\beta }_{0}+{\beta }_{1}{C}_{n}+{\beta }_{0i/C}+{\beta }_{1i/C}{C}_{n}+ {\varepsilon }_{i/C},$$

where ${\text{y}}_{\text{i}/\text{C}}$ = amplitude of the -ith participant (i) nested in condition (C), ${\upbeta }_{0}$ = fixed intercept, ${\upbeta }_{1}$ = fixed slope, ${\text{C}}_{\text{n}}$ = condition (n = face upright/face inverted), ${\upbeta }_{0\text{i}}$ = random intercept, ${\upbeta }_{1\text{i}/\text{C}}=$ random slope, ${\upvarepsilon }_{\text{i}/\text{C}}$ = overall variability.

#### Growth curve analysis (GCA)

We filtered the data from the earliest onset and the latest offset obtained from the BCPA, and applied GCA to that segment. First, GCA focuses on temporal profiles rather than solely on overall/average differences among groups, extending the method’s ability to detect differences as compared, for example, to traditional ANOVA^14^. Second, we used GCA utilising mixed regression models to examine how condition differences in amplitude change over time (i.e., the trajectory) while accounting for individual differences, accommodating the hierarchical structure of time-series data, where repeated measurements are nested within individuals and incorporated in random effects for individual subjects^71–74^. In turn, fixed effects enable the exploration of between-subject variations in the temporal profiles of amplitude condition differences. The advantage of this approach is that while fixed effects are used to examine between-group differences, individual variability is accounted for with random intercepts and slopes by participant. In other words, the fixed effect estimates are based on baselines and trajectories of individual participants, rather than of a group (e.g., males and females, that may be imbalanced). This accounts for potential biases (e.g., arising from differences in sample size between males and females) by modelling individual-specific patterns of change over time^74^.

We choose to run GCA separately by age-group (children, 6–11 years, adolescents, 12–17 yeas, and adults, > 18 years) to avoid a 4th-way interaction between the GCA defining polynomials, sex, neurotype and age (either as a continuous or an ordinal variable), but still being able to discern differences between age-groups. Each GCA by age-group took the amplitude difference between conditions as the dependent variable, 3rd degree orthogonal polynomials (linear, quadratic, and cubic) in interaction with neurotype and sex as fixed effects, and correlated random intercept per participants and slopes up to the 2nd order polynomials (Eq. 2).

2 $${y}_{i}= {\beta }_{0}+{\beta }_{1}{P}_{n}{x}_{i}\times {N}_{n}\times {S}_{n}+{\beta }_{0i}+{\beta }_{1i}{P}_{n}{x}_{i}+ {\varepsilon }_{i}.$$

${\text{y}}_{\text{i},\text{s}}$ = Condition Difference of the -ith participant (i), ${\beta }_{0}$ = fixed intercept, ${\beta }_{1}$ = fixed slope, ${{\text{P}}_{\text{n}}}{{\text{x}}_{\text{i}}}$ = polynomial function, ${\text{N}}_{\text{n}}$ = neurotype, ${\text{S}}_{\text{n}}$ = sex, ${\beta }_{0\text{i}}$ = random intercepts, ${{\beta }_{1\text{i}}}{{\text{P}}_{\text{n}}}{{\text{x}}_{\text{i}}}=$ random polynomials, ${\varepsilon }_{\text{i}}$ = overall variability.

For each model and significant effect, we report in the text the coefficient estimate, standard error, and p-value. The interpretation of these values and their implications for inferences is the same as any regression model (e.g., coefficient estimates equivalent to unstandardised effects size, and p-value indicating whether a specific factor, e.g., sex, influences the dependent variable, i.e., the amplitude condition difference). For each model, we also report coefficient estimates, standard error, and p-value of all effects in tables; in the table, we also report the T-value, a ratio between the coefficient estimate and the standard error (i.e., standardised), as to illustrate the distance of the coefficient estimate from zero or no effect^75^.

#### Exploratory pairwise correlations

We performed pairwise Spearman partial correlation by sex and neurotype between the individual random face-inversion effects obtained from the GCA, and FSIQ/SRS-2T-Score/ADOS CSS/the eye-tracking individual random effects obtained from the models reported in Ref.^10^, controlling for the age of the participant. The individual random effects represent the deviance of each individual from the estimated coefficients of each polynomial term, essentially reflecting individual trajectories. After fitting the GCA with Mixed Regression Models, these random effects can be directly accessed from the model object and extracted into a dataset for subsequent analysis^71^. We applied Bonferroni Correction to adjust the p-values of the correlations^76^. Specifically, we divided the significance threshold by 3, which accounts for the number of correlations conducted for each independent hypothesis (with Intercept, Slope, and Quadratic).

### Supplementary Information

Supplementary Information.

## Data Availability

The LEAP data is stored at a central database hosted by the Institut Pasteur (Paris, France) to efficiently and safely share the data ensuring participant confidentiality, quality control steps, pre-processing and download for registered analysis projects. To access the data used in this paper and/or receive a link to submit and register an analysis project, please email redcap@pasteur.fr. Detailed description and links to the study protocol are available in the LEAP protocol paper^55^.
