# Supplementary material for: Sex differences in social brain neural responses in autism: temporal profiles of configural face-processing within data-driven time windows
Source: Sci Rep. 2024 Jun 18;14:14038. doi: 10.1038/s41598-024-64387-9 (PMC11189412; doi:10.1038/s41598-024-64387-9)
Supplement: Supplementary file 1 — Supplementary Information. [file 41598_2024_64387_MOESM1_ESM.pdf]

“Sex Differences in Social Brain Neural Responses in Autism: Temporal Profiles of Configural Face-Processing Within Data-Driven Time Windows”
Supplementary Material (SM)

Authors:

Teresa Del Bianco[1,2]*, Meng-Chuan Lai[3,4,5,6,7], Luke Mason[1,8], Mark H. Johnson[9], Tony Charman[8], Eva Loth[8], Tobias Banaschewski[10], Jan Buitelaar[11], Declan G. M. Murphy[8], Emily J. H. Jones[1] and the AIMS-2-TRIALS LEAP Team[12]

1. Centre for Brain and Cognitive Development, Birkbeck University of London, London, United Kingdom
2. School of Social Sciences And Professions, London Metropolitan University, London, United Kingdom
3. Margaret and Wallace McCain Centre for Child, Youth & Family Mental Health and Azrieli Adult Neurodevelopmental Centre, Campbell Family Mental Health Research Institute, Centre for Addiction and Mental Health, Toronto, Ontario, Canada
4. Department of Psychiatry, Temerty Faculty of Medicine, University of Toronto, Toronto, Ontario, Canada
5. Department of Psychiatry, The Hospital for Sick Children, Toronto, Ontario, Canada
6. Autism Research Centre, Department of Psychiatry, University of Cambridge, Cambridge, United Kingdom
7. Department of Psychiatry, National Taiwan University Hospital and College of Medicine, Taipei, Taiwan
8. Institute of Psychiatry, Psychology & Neuroscience, King’s College London, London, United Kingdom
9. Department of Psychology, University of Cambridge, Cambridge, United Kingdom
10. Central Institute of Mental Health, Mannheim, Germany

11. Donders Center of Medical Neurosciences, Radboud University, Nijmegen, The Netherlands
12. The AIMS-2-TRIALS LEAP Team comprises: Simon Baron-Cohen, Sarah Durston, Antonio Persico, Sven Bölte, Antonia San Jose Caceres, Hannah Hayward, Daisy Crawley, Jessica Faulkner, Jessica Sabet, Claire Ellis, Bethany Oakley, Rosemary Holt, Sara Ambrosino, Nico Bast, Sarah Baumeister, Annika Rausch, Carsten Bours, Ineke Cornelissen, Daniel von Rhein, Larry O’Dwyer, Julian Tillmann, Jumana Ahmad, Emily Simonoff, Carsten Bours, Joerg Hipp, Pilar Garces, Christine Ecker, Andreas Meyer-Lindenberg, Heike Tost, Carolin Moessnang, Daniel Brandeis, Christian Beckmann, Flavio Dell’ Acqua, Robert Dallyn, Cate Davidson, Amber Ruigrok, Thomas Bourgeron, Claire Leblond

* Contact Information: Centre for Brain and Cognitive Development, Henry Wellcome Building, Birkbeck, University of London, Malet Street, London, WC1E 7HX. Email: t.delbianco@bbk.ac.uk

Topography of Amplitudes **.......... 4**

Bootstrapped Cluster-based Permutation Analysis **.......... 4**

Replication of Bootstrapped Cluster-based Permutation Analysis on Averaged Electrode Clusters **.......... 5**

Electrode Cluster 1 (P8, PO8 and TP10) 5
Electrode Cluster 2 (PO2, POZ and PO4) 6
Electrode Cluster 3 (P8, P7, PO7, and PO8) 7

Goodness of Fit of Cubic Polynomial Order **.......... 7**

Growth Curve Analysis Random Effects Estimates **.......... 8**

Replication of Growth Curve Analysis on subset with Attention Coding **.......... 9**

Exploratory Pairwise Correlations **.......... 13**

FSIQ 13
SRS-2 14
ADOS CSS Total 14
Eye-Tracking Face Pop Out 14
Eye-Tracking Dynamic Video 15

Topography of Amplitudes

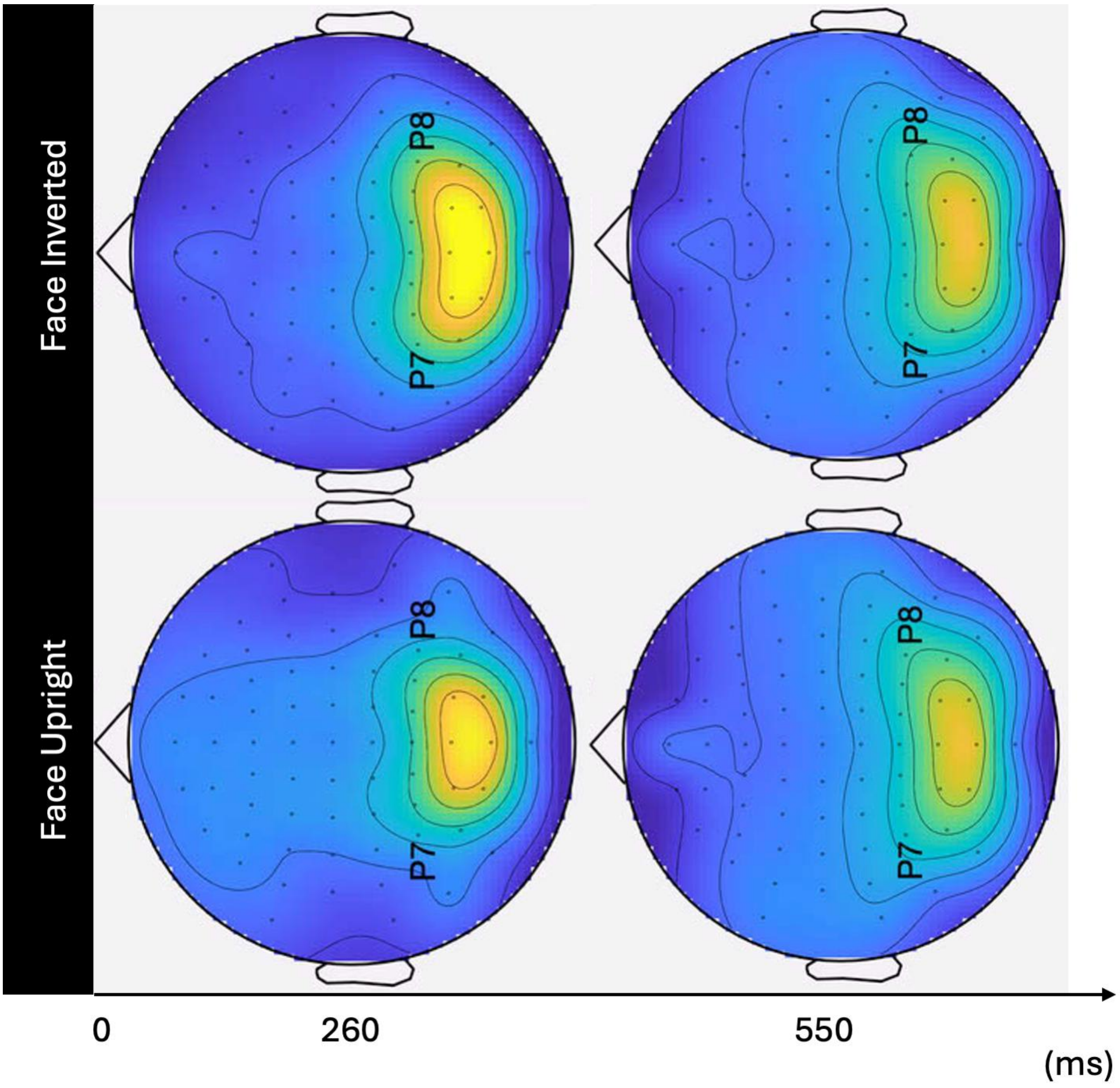

Figure S 1: Topographical distribution of averaged amplitudes at 250 ms and 550 ms post-stimulus, with positions of P8 and P7.

Bootstrapped Cluster-based Permutation Analysis

Below we report the full list of beta coefficients, standard errors, and p-values obtained from each of the non-parametric bootstrapping.

Table S 1: beta (standardised) coefficients, standard errors and p-values for each of the onset and offsets detected by the analysis.

Subgroup	Effect	Value	Time	Beta	SE	P-Value
All	Group * Condition	Onset	310	0.17	0.08	0.02
		Offset	390	0.16	0.07	0.01
	Sex * Condition	Onset	260	-0.29	0.03	0.01
		Offset	440	-0.004	0.00009	0.04
Female	Group * Condition	Onset	430	-1.05	1.29	< 0.001
		Offset	480	-0.9	1.17	< 0.001
Male	Group * Condition	Onset	310	0.83	0.98	< 0.001
		Offset	450	0.67	0.90	< 0.001
Autistic	Sex * Condition	Onset	260	-0.94	0.88	0.01
		Offset	550	-0.41	0.38	< 0.001
Neurotypical	Sex * Condition	Onset	280	-0.75	0.62	< 0.001
		Offset	370	-0.79	0.81	< 0.001

Replication of Bootstrapped Cluster-based Permutation Analysis on Averaged Electrode Clusters

We averaged amplitudes across two clusters of electrodes:

(1) P8 with PO8 and TP10
(2) PO2, POZ and PO4
(3) P8, P7, PO7, PO8

We entered the averaged amplitude as dependent variable of the BCPA following Equation 1 of the main text.

Electrode Cluster 1 (P8, PO8 and TP10)

The BCPA did not find a time-frame where the condition by group interaction was significant, but it did find a time-frame where the condition by sex interaction was significant, between 260 - 580 ms (beta = -1.10, SE = 1.80, p = <0.001). This time-frame overlaps with the time-frame where sex differences were significant at P8 (260 - 440).

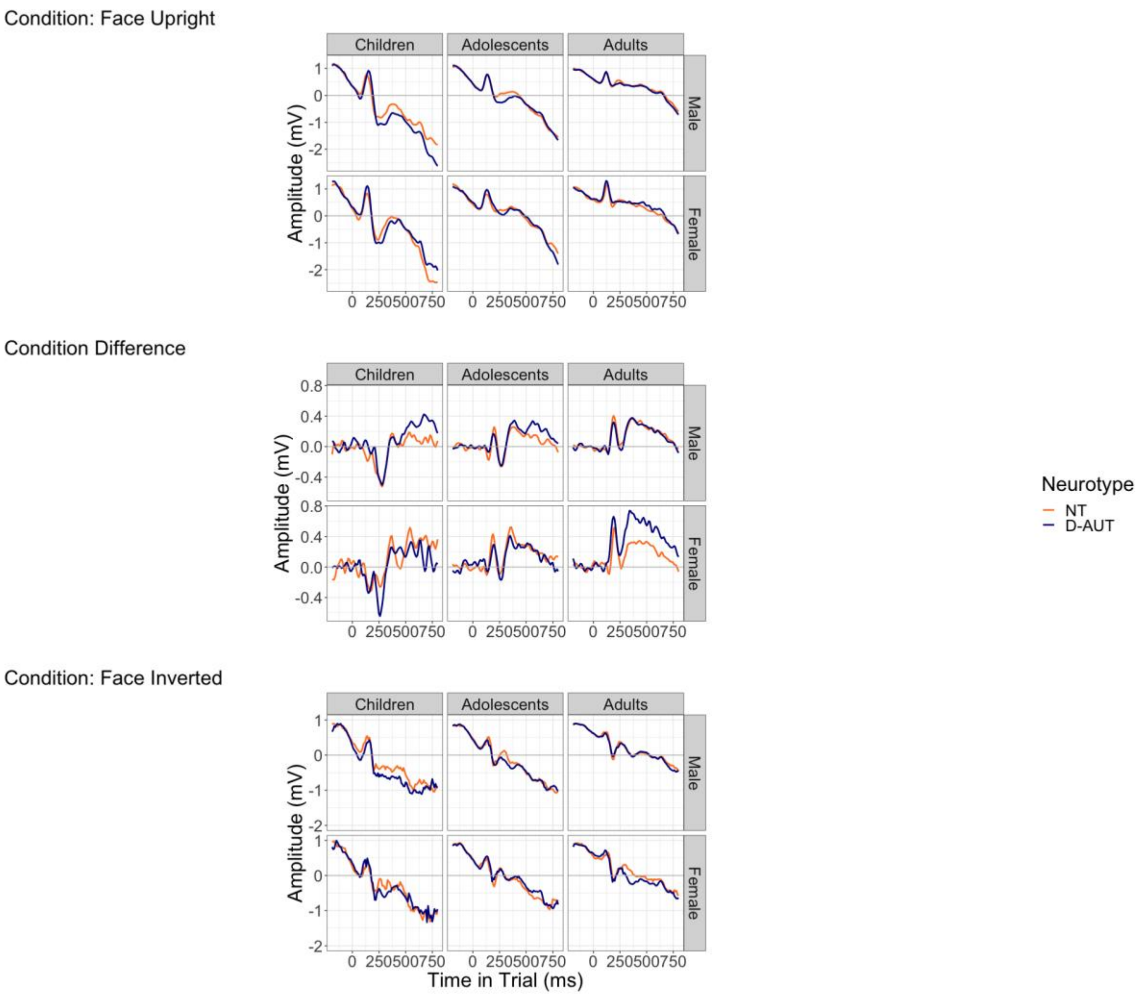

Figure S 2: amplitudes in the two conditions, and subtracted from each other (middle) from averaging P8, PO8 and TP10

Electrode Cluster 2 (PO2, POZ and PO4)

The BCPA did not find a time-frame where the condition by group interaction was significant, but it did find a time-frame where the condition by sex interaction was significant, between 270 - 380 ms (beta = -0.91, SE = 1.23, p = 0.001). This time-frame overlaps with the time-frame where sex differences were significant at P8 (260 - 440).

In summary, the averaged clusters of electrodes seemed to be equally sensitive to condition differences between males and females, but not between autistic and non-autistic (see Figure SX). This may be due to larger variability between conditions due to averaging, as indexed by the larger standard errors and the plots below.

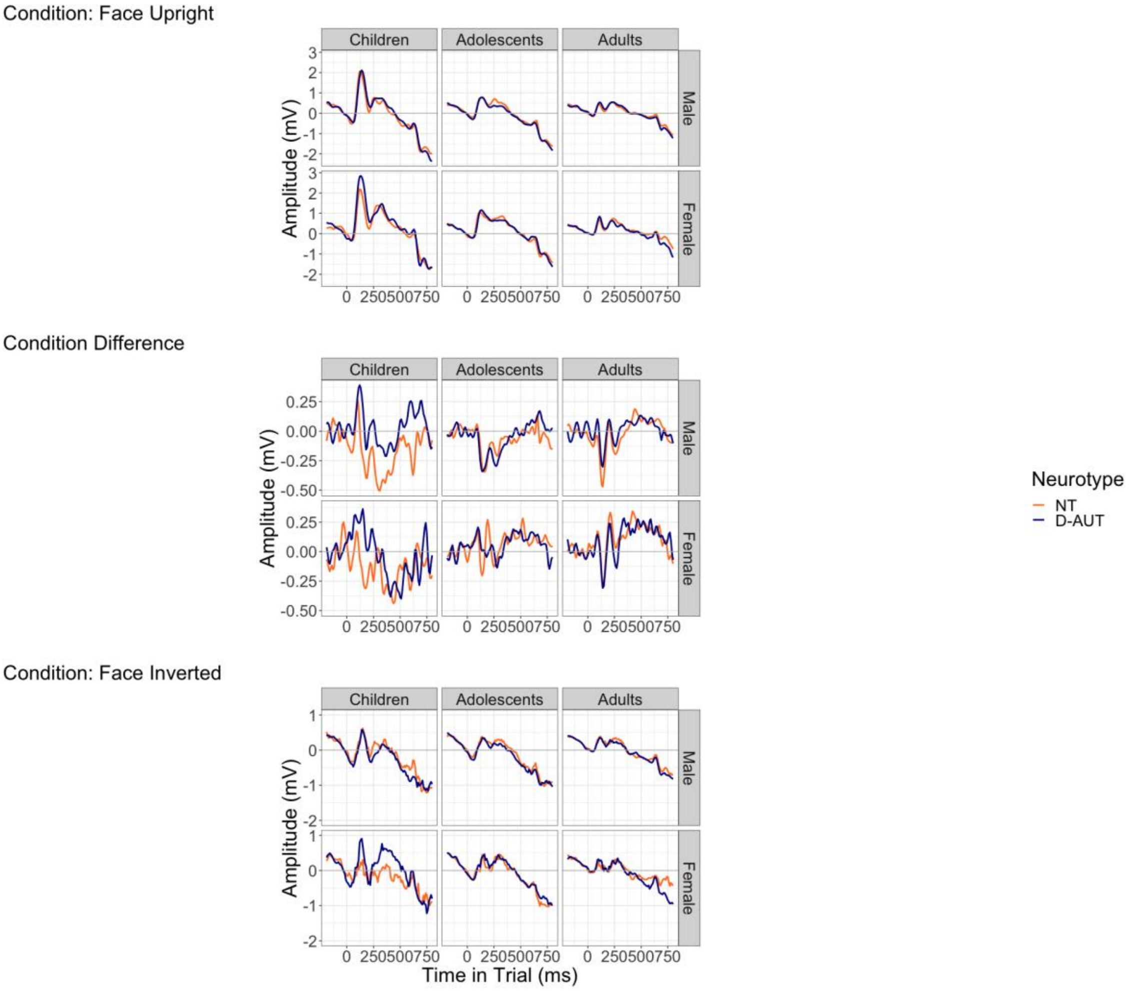

Figure S 3: amplitudes in the two conditions, and subtracted from each other (middle) from averaging PO2, POZ and PO4

Electrode Cluster 3 (P8, P7, PO7, and PO8)

The BCPA did not find a time-frame where the condition by group interaction was significant, but it did find a time-frame where the condition by sex interaction was significant, between 170 640 ms (beta = -1.23, SE = 2.14, p = <0.001). This time-frame includes the time-frame where sex differences were significant at P8 only (260 - 440).

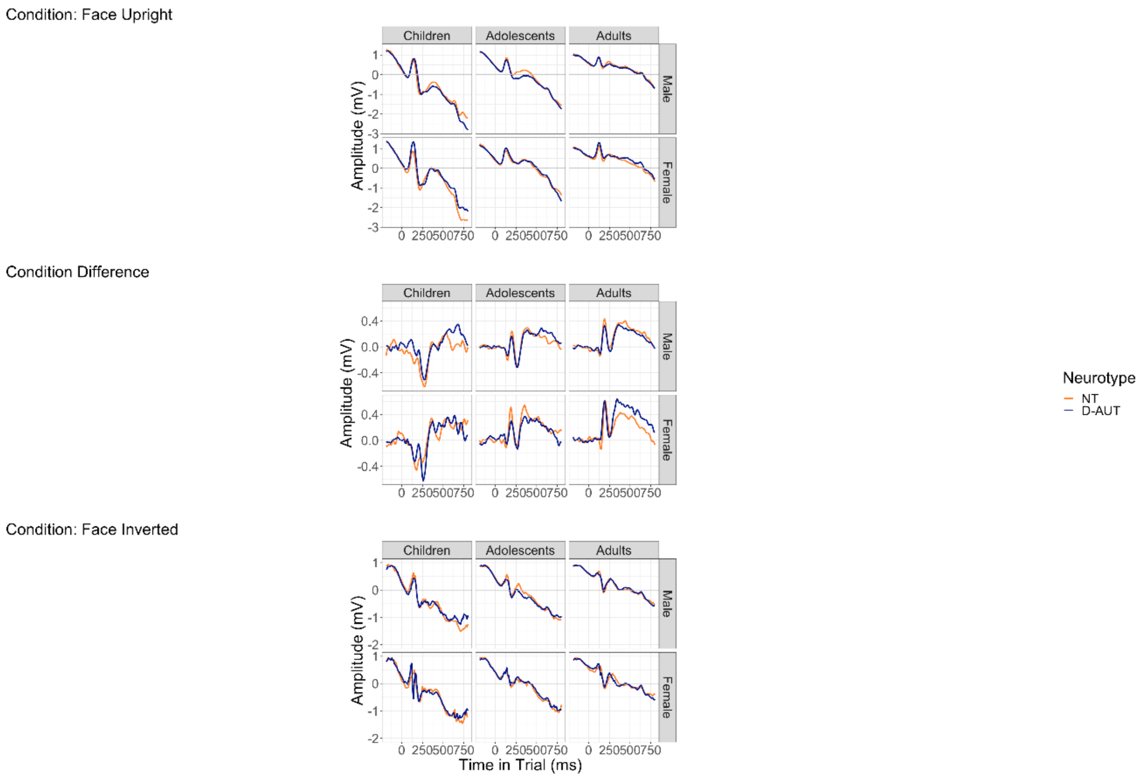

Figure S 4: amplitudes in the two conditions, and subtracted from each other (middle) from averaging PO2, POZ and PO4

Goodness of Fit of Cubic Polynomial Order

We compared a linear, quadratic and cubic fit with the Likelihood Ratio test (LRT). As shown from the smaller AIC, BIC and deviance, and the significant p-values, the cubic fit is the best fit to all models.

Table S 2: goodness of fit of polynomial orders form linear to cubic on the children subset

Order	Npar	AIC	BIC	deviance	p-value
1st (linear)	12	6758.83	6829.91	6734.83	-
2nd (quadratic)	19	6604.47	6717.01	6566.47	<0.001
3rd (cubic)	23	5838.21	5974.44	5792.21	<0.001

Table S 3: Table SX: goodness of fit of polynomial orders form linear to cubic on the adolescents subset

Order	Npar	AIC	BIC	deviance	p-value
1st (linear)	12	10403.95	10483.48	10379.95	-
2nd (quadratic)	19	9013.05	9138.05	8975.05	<0.001

3rd (cubic)	23	8280.23	8432.65	8234.23	<0.001

Table S 4: goodness of fit of polynomial orders form linear to cubic on the adults subset

Order	Npar	AIC	BIC	deviance	p-value
1st (linear)	12	7619.14	7698.92	7595.14	-
2nd (quadratic)	19	5941.02	6067.33	5903.02	<0.001
3rd (cubic)	23	5557.40	5710.31	5511.40	<0.001

Growth Curve Analysis Random Effects Estimates

Table S 5: estimate of the variance and correlation of random effects for each component for the GCA ran in the Children

effect	grouping	term	estimate
random	Participant	Intercept	3.98
random	Participant	Intercept/Linear Component (Slope)	-0.95
random	Participant	Correlation: Intercept/Quadratic Component	0.97
random	Participant	Linear Component (Slope)	29.84
random	Participant	Correlation: Linear Component (Slope)/Quadratic Component	-0.89
random	Participant	Quadratic Component	35.86
random	Residual		0.60

Table S 6: estimate of the variance and correlation of random effects for each component for the GCA ran in the Adolescents

effect	grouping	term	estimate
random	Participant	Intercept	2.92
random	Participant	Correlation: Intercept/Linear Component (Slope)	-0.96
random	Participant	Intercept/Quadratic Component	0.97
random	Participant	Linear Component (Slope)	22.37

random	Participant	Correlation: Linear Component (Slope)/Quadratic Component	-0.90
random	Participant	Quadratic Component	24.60
random	Residual	Observation	0.44

Table S 7: estimate of the variance and correlation of random effects for each component for the GCA ran in the Children

**effect**	**grouping**	**term**	**Coefficient estimate**
random	Participant	Intercept	2.32
random	Participant	Intercept/Linear Component (Slope)	-0.96
random	Participant	Intercept/Quadratic Component	0.97
random	Participant	Linear Component (Slope)	17.66
random	Participant	Correlation: Linear Component (Slope)/Quadratic Component	-0.91
random	Participant	Quadratic Component	20.29
random	Residual	Observation	0.34

Replication of Growth Curve Analysis on subset with Attention Coding

To disentangle the effects of the factor of interest (group, age and gender) and the level of attentiveness, we have replicated the GCA on the subset of participants from the site King's College London that had manual online coding of attention available (N = 292). We received this measure aggregated as a categorical variable with two levels (=/>90% attentiveness, and <90% attentiveness). We assumed this variable as a covariate in the replicated models.
We found that the cubic component of the GCA significantly interacted with group and sex in the children, meaning that amplitude was more negative for upright faces earlier, followed by more positive and then negative amplitudes as in the whole children sample. This difference did not turn out significant in the adolescent and adult subgroups with attention coding. The level of attentiveness did not turn out significant in any of the models. Overall, this indicates that the pattern found in the whole sample without attention coding is preserved, and excludes that results observed in children may be explained by lower levels of attentiveness. Furthermore, the non-significant covariate excludes that the loss of significance of the effect in adolescents may be due to attentiveness, but more likely due to smaller sample (total = 122 vs 186).

Table S 8: coefficients, standard errors (SE), t-values, degrees of freedom (df), p-values of the GCA run on the Children subset

term	Coefficient estimate	SE	T-Value	df	P-value
(Intercept)	-0.14	0.32	70.66	-0.43	0.66
Linear Component (Slope)	-29.77	3.45	778.51	-8.60	<0.001
Quadratic Component	1.07	3.26	54.33	0.32	0.74
Cubic Component	-22.42	2.03	3650	-11.02	<0.001
Neurotype (Autistic)	0.44	0.29	56.58	1.52	0.13
Sex (Female)	0.41	0.35	56.46	1.16	0.24
Level of Attentiveness (>90%)	-0.12	0.26	52.99	-0.46	0.64
Linear Component (Slope) * Neurotype (Autistic)	14.05	4.29	778.51	3.27	0.001
Quadratic Component * Neurotype (Autistic)	9.39	4.05	54.33	2.31	0.02
Cubic Component * Neurotype (Autistic)	9.68	2.52	3650.	3.83	0.00012
Linear Component (Slope) * Sex (Female)	33.69	5.24	778.51	6.42	<0.001
Quadratic Component * Sex (Female)	1.92	4.95	54.33	0.38	0.69
Cubic Component * Sex (Female)	24.78	3.08	3650	8.03	<0.001
Neurotype (Autistic) * Sex (Female)	-0.59	0.47	56.44	-1.25	0.21
Linear Component (Slope) * Neurotype (Autistic) * Sex (Female)	-43.73	6.93	778.51	-6.30	<0.001

Quadratic Component * Neurotype (Autistic) * Sex (Female)	-8.96	6.55	54.33	-1.36	0.17
Cubic Component * Neurotype (Autistic) * Sex (Female)	-32.45	4.08	3650	-7.95	<0.001

Table S 9: coefficients, standard errors, t-values, DF, p-values of the GCA run on the Adolescents subset. Significant coefficients are marked with ''*

term	Coefficient estimate	SE	T-Value	df	P-value
(Intercept)	-0.27	0.15	153.64	-1.85	0.06
Linear Component (Slope)	-18.61	1.57	1974.44	-11.80	<0.001
Quadratic Component	-2.02	1.58	118.67	-1.27	0.20
Cubic Component	-14.61	0.94	7682	-15.52	<0.001
Neurotype (Autistic)	0.17	0.11	127.85	1.46	0.14
Sex (Female)	0.23	0.15	128.51	1.53	0.12
Level of Attentiveness (>90%)	-0.02	0.13	116.99	-0.21	0.83
Linear Component (Slope) * Neurotype (Autistic)	0.50	2.04	1974.44	0.24	0.80
Quadratic Component * Neurotype (Autistic)	2.64	2.05	118.67	1.28	0.20
Cubic Component * Neurotype (Autistic)	0.61	1.22	7682	0.50	0.61
Linear Component (Slope) * Sex (Female)	3.24	2.63	1974.44	1.23	0.21
Quadratic Component * Sex (Female)	-1.23	2.64	118.67	-0.46	0.64

Cubic Component * Sex (Female)	2.46	1.57	7682	1.57	0.11
Neurotype (Autistic) * Sex (Female)	-0.07	0.20	128.43	-0.38	0.70
Linear Component (Slope) * Neurotype (Autistic) * Sex (Female)	0.34	3.61	1974.44	0.09	0.92
Quadratic Component * Neurotype (Autistic) * Sex (Female)	1.02	3.63	118.67	0.281	0.77
Cubic Component * Neurotype (Autistic) * Sex (Female)	-1.18	2.15	7681.99	-0.55	0.58

Table S 10: coefficients, standard errors, t-values, DF, p-values of the GCA run on the Adult subset. Significant coefficients are marked with ''*

term	Coefficient estimate	SE	T-Value	df	P-value
(Intercept)	-0.18	0.10	137	-1.76	0.079
Linear Component (Slope)	-10.84	1.34	1759	-8.09	<0.001
Quadratic Component	-3.93	1.16	106.80	-3.36	0.001
Cubic Component	-9.43	0.79	6926	-11.80	<0.001
Neurotype (Autistic)	-3.51	0.09	114.60	-0.37	0.70
Sex (Female)	0.08	0.13	114.60	0.67	0.50
Level of Attentiveness (>90%)	0.0007	0.08	1050	0.009	0.99
Linear Component (Slope) * Neurotype (Autistic)	-1.82	1.71	1759	-1.06	0.28

Quadratic Component * Neurotype (Autistic)	0.31	1.49	106.8	0.21	0.83
Cubic Component * Neurotype (Autistic)	-0.81	1.02	6926	-0.79	0.42
Linear Component (Slope) * Sex (Female)	-6.16	2.43	1759	-2.53	0.01
Quadratic Component * Sex (Female)	1.76	2.12	106.8	0.82	0.40
Cubic Component * Sex (Female)	-3.76	1.45	6926	-2.59	0.009
Neurotype (Autistic) * Sex (Female)	-0.13	0.16	114.6	-0.80	0.42
Linear Component (Slope) * Neurotype (Autistic) * Sex (Female)	4.43	3.12	1759	1.41	0.15
Quadratic Component * Neurotype (Autistic) * Sex (Female)	-4.68	2.72	106.8	-1.71	0.08
Cubic Component * Neurotype (Autistic) * Sex (Female)	2.31	1.86	6926	1.24	0.21

Exploratory Pairwise Correlations

From the base models (i.e., excluding the grouping factors – group and sex) we extracted individual coefficients for the random intercept and slopes (linear and quadratic polynomial) to explore correlations with (1) clinical measures of interest: Full Scale IQ (FSIQ), Social Responsiveness Scale (SRS-2), and the Autism Diagnostic Observation Schedule (ADOS) calibrated total score (2) the eye-tracking individual coefficients used in (Del Bianco et al., 2022). The obtained p-values were Bonferroni-corrected for multiple comparisons based on 3 hypothesis (that each variable has a relationship with the (1) intercept, (2) slope and (3) quadratic component of the GCA, and thus influence the average amplitude, rate of change and shape of the face inversion effect). Below we report the full list of Spearman correlation coefficients (rho) and p-values.

FSIQ

We applied Bonferroni Correction to adjust the p-values. Specifically, we multiplied the p-values by 3, which accounts for the number of correlations conducted for each independent hypothesis (with Intercept, Slope, and Quadratic).

Table S 11: correlation coefficients (rho) and Bonferroni-corrected p-values for each combination of variables. Significant coefficients are marked with ''*

Neurotype		Autistic		Neurotypical	
Sex		Female	Male	Female	Male
N		71	187	74	133
Rho	Intercept	-0.18	0.15	0.32	0.10
P-value		0.40	0.14	0.02*	0.74
Rho	Slope	0.15	-0.11	-0.36	-0.08
P-value		0.66	0.41	0.01*	1.02
Rho	Quadratic	-0.11	0.15	0.25	0.09
P-value		1.05	0.13	0.09	0.92

SRS-2

We applied Bonferroni Correction to adjust the p-values. Specifically, we multiplied the p-values by 3, which accounts for the number of correlations conducted for each independent hypothesis (with Intercept, Slope, and Quadratic).

Table S 12: correlation coefficients (rho) and Bonferroni-corrected p-values for each combination of variables. Significant coefficients are marked with ''*

Neurotype		Autistic		Neurotypical	
Sex		Female	Male	Female	Male
N		68	170	65	107
Rho	Intercept	0.13	-0.01	-0.20	-0.08
P-value		0.88	2.62	0.34	1.23
Rho	Slope	-0.12	-0.03	0.26	0.02
P-value		1.00	2.16	0.11	2.56
Rho	Quadratic	0.09	-0.04	-0.12	-0.01
P-value		1.44	1.77	1.01	2.81

ADOS CSS Total

We applied Bonferroni Correction to adjust the p-values. Specifically, we multiplied the p-values by 3, which accounts for the number of correlations conducted for each independent hypothesis (with Intercept, Slope, and Quadratic).

Table S 13: correlation coefficients (rho) and Bonferroni-corrected p-values for each combination of variables. Significant coefficients are marked with ''*

Neurotype		Autistic	D-AUT
Sex		Female	Male
N		72	183
Rho	Intercept	0.15	0.01
P-value		0.64	2.63
Rho	Slope	-0.11	-0.02
P-value		1.10	2.29
Rho	Quadratic	0.14	0.03
P-value		0.68	1.93

Eye-Tracking Face Pop Out

We applied Bonferroni Correction to adjust the p-values. Specifically, we multiplied the p-values by 3, which accounts for the number of correlations conducted for each independent hypothesis (with Intercept, Slope, and Quadratic).

Table S 14: correlation coefficients (rho) and Bonferroni-corrected p-values for each combination of variables. Significant coefficients are marked with ''*

Neurotype		Autistic		Neurotypical	
Sex		Female	Male	Female	Male
N		64	166	66	120
ET Intercept - Rho	Intercept	0.11	0.10	0.17	0.02
P-value		1.18	0.62	0.49	2.58
ET Intercept - Rho	Slope	-0.03	-0.13	-0.21	-0.04
P-value		2.52	0.31	0.28	1.91
ET Intercept - Rho	Quadratic	0.21	0.09	0.10	0.02
P-value		0.30	0.78	1.31	2.56
ET Slope - Rho	Intercept	0.13	-0.03	0.10	0.00
P-value		0.98	2.14	1.34	2.89
ET Slope - Rho	Slope	-0.07	0.00	-0.11	0.02

P-value		1.79	2.86	1.17	2.55
ET Slope - Rho	Quadratic	0.11	-0.06	-0.01	0.01
P-value		1.12	1.40	2.80	2.79
ET Quadratic - Rho	Intercept	-0.03	-0.05	0.20	-0.05
P-value		2.51	1.53	0.35	1.73
ET Quadratic - Rho	Slope	0.05	0.13	-0.23	0.02
P-value		2.17	0.27	0.21	2.50
ET Quadratic - Rho	Quadratic	0.05	0.13	-0.23	0.02
P-value		2.17	0.27	0.21	2.50

Eye-Tracking Dynamic Video

We applied Bonferroni Correction to adjust the p-values. Specifically, we multiplied the p-values by 3, which accounts for the number of correlations conducted for each independent hypothesis (with Intercept, Slope, and Quadratic).

Table S 15: correlation coefficients (rho) and Bonferroni-corrected p-values for each combination of variables. Significant coefficients are marked with ''*

Neurotype		Autistic		Neurotypical	
Sex		Female	Male	Female	Male
N		62	168	63	119
ET Intercept - Rho	Intercept	0.12	0.07	0.15	0.06
P-value		1.09	1.09	0.71	1.56
ET Intercept - Rho	Slope	-0.09	-0.03	-0.16	-0.07
P-value		1.51	2.05	0.61	1.31
ET Intercept - Rho	Quadratic	0.10	0.07	0.08	0.01
P-value		1.30	1.01	1.66	2.86
ET Slope - Rho	Intercept	0.13	0.03	0.12	0.06
P-value		0.92	2.01	1.06	1.61
ET Slope - Rho	Slope	-0.10	0.00	-0.13	-0.07
P-value		1.34	2.91	0.99	1.27
ET Slope - Rho	Quadratic	0.11	0.03	0.04	0.00

P-value		1.14	2.09	2.21	2.99
ET Quadratic - Rho	Intercept	0.13	0.03	0.12	0.06
P-value		1.07	0.05	0.32	1.70
ET Quadratic - Rho	Slope	0.09	0.15	0.23	0.06
P-value		1.40	0.18	0.22	1.59
ET Quadratic - Rho	Quadratic	0.09	0.15	0.23	0.06
P-value		1.40	0.18	0.22	1.59
